# Anti-Drug Antibodies, Drug Levels, Interleukin-6 and Soluble TNF Receptors in Rheumatoid Arthritis Patients during the First 6 Months of Treatment with Adalimumab or Infliximab: A Descriptive Cohort Study

**DOI:** 10.1371/journal.pone.0162316

**Published:** 2016-09-08

**Authors:** Grith Petersen Eng, Pierre Bouchelouche, Else Marie Bartels, Henning Bliddal, Klaus Bendtzen, Michael Stoltenberg

**Affiliations:** 1 Department of Clinical Biochemistry, Zealand Universityhospital Køge, Køge, Denmark; 2 Department of Rheumatology, Zealand Universityhospital Køge, Køge, Denmark; 3 The Parker Institute, Department of Rheumatology, Copenhagen University Hospital, Bispebjerg and Frederiksberg, Denmark; 4 Institute for Inflammation Research, Copenhagen University Hospital, Rigshospitalet, Denmark; Keio University, JAPAN

## Abstract

**Objectives:**

With the present study we wanted to explore the impact of treatment with a tumor necrosis factor-α -inhibitor (TNFi) on levels of soluble biomarkers in rheumatoid arthritis (RA) patients and to identify predictors of impaired drug levels and development of anti-TNFi antibodies (anti-TNFi Abs).

**Methods:**

Blood samples from 26 patients with established RA were taken at baseline and following 6 months of treatment with adalimumab or infliximab. Samples were analyzed for levels of TNFi, interleukin (IL)-6, and soluble TNF-receptors 1 and -2 (sTNF-R1 and -2) and for presence of anti-TNFi Abs. Clinical and demographic data were recorded as well.

**Results:**

During the initial 6 months treatment, DAS28(CRP) (Disease activity score in 28 joints using C-reactive protein) and levels of IL-6 and sTNF-R2 decreased significantly in patients without anti-TNFi Abs and in patients retaining detectable drug levels. The levels of other tested cytokines (TNF-α, TNF-β, IL-1ra, IL-1b, IL-8, IL-10, IL-12(p70), IL-13, IL-17A, IL-17F, and IL-33) were generally below detection limits. Higher baseline levels of IL-6 associated with undetectable levels of TNFi at follow-up. Anti-TNFi Abs were associated with decreased drug levels, but no predictors for anti-TNFi Ab development could be found.

**Conclusion:**

The effect of treatment with TNFi on RA disease activity depends on levels of active drug, and by presence of anti-TNFi Abs. In patients who retain detectable drug levels, and in the absence of anti-TNFi Abs, clinical outcome is improved during treatment, and circulating levels of IL-6 and sTNF-R2 decrease. Baseline levels of IL-6 may predict depletion of TNFi and may identify patients at risk of treatment failure.

## Introduction

Although biological TNF-α inhibitors (TNFi) have revolutionized the treatment of rheumatoid arthritis (RA) and other autoimmune inflammatory diseases, only one third of RA patients will experience a sustained treatment response [1–3]. Higher serum concentrations of TNFi are associated with better clinical outcome, whereas anti-TNFi antibodies (Abs) are associated with infusion reactions and treatment failure. As yet, efforts to identify other predictors of treatment response have had little success [4–8], although higher baseline levels of IL-6 may predict efficacy of infliximab treatment [9].

Anti-TNFi Abs may develop in patients treated with a TNFi, and their presence in the patient is associated with lower levels of bioactive TNFi, impaired clinical efficacy, and adverse reactions [6;10–14]. Anti-TNFi Abs are more often detected in patients treated with adalimumab (Humira®) and infliximab (Remicade®) than in patients treated with other available TNFi [13]. It is, however, still unknown why some patients develop anti-TNFi Abs, while others do not. Patients developing anti-TNFi Abs most often do so within the first 6–12 months of treatment, but measurable anti-TNFi Abs may also develop after several years of treatment [11].

While TNFi selectively target TNF-α and, in case of etanercept, TNF-β as well, the secondary effects on the various components of the immune system are only vaguely understood [15]. However, interleukin (IL)-6, another pro-inflammatory cytokine, also appears to be a key cytokine in the inflammatory cascade fuelling inflammation in RA [16]. Thus, IL-6 is elevated in serum and synovia of patients with active RA, and decreases in responders to TNFi therapy [15;17;18].

The soluble TNF-α receptors 1 and 2 (sTNF-R1 and sTNF-R2) are extracellular products of enzymatic cleavage of membrane-bound TNF-R1 and TNF-R2. These naturally occurring extracellular TNF-receptors are known to down-regulate TNF-α activity, thus counteracting inflammatory responses initiated by TNF-α. Both sTNF-R1 and -R2 are elevated in patients with RA, and sTNF-R2 is known to correlate with disease activity in RA and in other inflammatory diseases [19;20].

To identify easily obtainable biomarkers, which might predict the development and therapeutic consequences of anti-TNFi Ab, we investigated early appearance of circulating anti-TNFi Ab and corresponding levels of TNFi in RA patients, and related the anti-TNFi Ab and drug levels to serum levels of IL-6, sTNF-R1 and sTNF-R2.

## Patients and Methods

### Study design and population

The study was conducted in accordance with the Helsinki Declaration (www.wma.net/en/30publications/10policies/b3/) and approved by the local ethics committee (KF 01-045/03), and written informed consent was obtained from all patients prior to inclusion. The patients participating in this study were recruited from a cohort with established RA according to the American College of Rheumatology (ACR) 1987 criteria [21]. All started treatment with a biological TNFi during the period 2003–2007 at the outpatient clinic at the University Hospital of Copenhagen, Frederiksberg, Denmark, as earlier described in detail [22]. Patients were monitored at baseline and at follow-up, including clinical examination, blood sampling for experimental and para-clinical measures, disease activity score in 28 joints and with the use of C-reactive protein (DAS28(CRP)), and imaging in the form of ultrasound and magnetic resonance imaging (MRI). In this cohort, the choice of TNF-α inhibitor was at the treating physician’s discretion. The patients included in the study received standard dose and treatment interval of TNFi (Adalimumab 40 mg s.c. /2 weeks, Infliximab induction regimen of 3 mg/kg at weeks 0, 2, 6 and then 3 mg/kg every 8 weeks. In the present study, patients treated with adalimumab or infliximab who sustained therapy for at least 6 months were selected. Only patients with adequate baseline and 6-month follow-up blood samples were included. All experimental laboratory analyses were carried out in a blinded manner.

### Laboratory analysis

Baseline and follow-up blood samples were taken in the morning, immediately before administration of TNFi. All patients were fasting at sampling. Without delay, serum was produced and stored at -80° until assayed. Blood samples at scheduled 6-months follow-up also were taken on the day of the treatment, immediately before the planned administration of TNFi.

Immunoglobulin M rheumatoid factor (IgM-RF) was measured at Department of Clinical Biochemistry at Slagelse Hospital, Denmark, using immuno-turbidimetric analysis on Cobas 6000 c-501 (F. Hoffmann-La Roche Ltd., Hvidovre, Denmark). IgM-RF was considered positive at values above 14 IU/ml.

CRP was measured at the Department of Clinical Biochemistry, Copenhagen University Hospital, Frederiksberg, using immunometric analysis on Vitros 5.1FS (Ortho Clinical Diagnostics Inc., Raritan NJ, USA).

#### Concentrations of TNFi and anti-TNFi antibodies

The levels of bioactive adalimumab and infliximab were measured using reporter gene assays (RGAs) iLite^TM^ Infliximab Bioassay and iLite^TM^ Adalimumab Bioassay, respectively (Biomonitor, Copenhagen, Denmark), as previously described [23]. Detection limit was 0.7 μg/ml for both assays, and results below detection were set to the detection limit.

Anti-adalimumab and anti-infliximab antibodies were detected in serum using fluid-phase radioimmunoassay (RIA) (Biomonitor), as previously detailed [4]. Anti-TNFi Ab-positivity was defined as detectable levels (i.e., above the limit of quantification of 10 arbitrary units/ml of both anti-adalimumab and anti-infliximab antibodies).

#### Concentrations of IL-6, sTNF-R1 and sTNF-R2

IL-6, sTNF-R1, and sTNF-R2 were measured in serum using commercially available assays according to the manufacturers’ directions. The analyses were performed at the Institute for Inflammation Research, Copenhagen University Hospital, Rigshospitalet, Copenhagen, Denmark.

IL-6 was measured using a Bio-Plex Pro^TM^ Human Th17 Cytokine Assay (Bio-Rad Laboratories, Hercules, CA, USA) as part of a kit measuring a range of cytokines including IL-1β, IL-10, IL-17A, IL-17F, IL-33 and TNF-α. Test results were read using the Luminex 100 System (Luminex, Austin, TX, USA). The sensitivity of the assay for IL-6 was 2 pg/ml. Levels of IL-6 below the detection limit were set to the median value of 1 pg/ml. The sensitivity of the assay for the remainder of cytokines was 0.2 pg/ml for IL-1β, 11 pg/ml for IL-10, 3 pg/ml for IL-17A, 9 pg/ml for IL-17F, 5 pg/ml for IL-33, and 1 pg/ml for TNF-α. The samples also were tested for levels of IL-1ra, IL-8, IL-12(p70), IL-13 and TNF-β using Bio-Plex Pro^TM^ Human Cytokine Assay (Bio-Rad Laboratories). The sensitivity of this assay was 64 pg/ml for IL-1ra, 7 pg/ml for IL-8, 9 pg/ml for IL-12(p70), 10 pg/ml for IL-13 and 6 pg/ml for TNF- β.

sTNF-R1 and sTNF-R2 were measured using Quantikine immunoassays DRT100 and DRT200 (R&D Systems, Minneapolis, MN, USA). These assays are solid-phase enzyme linked immuno sorbent assays (ELISAs), and the sensitivities of the assays were 40 pg/ml and 16 pg/ml, respectively. The upper detection limit was 3750 pg/ml, and levels of sTNFR-R2 above detection were set to this upper detection limit.

### Statistics

Median and interquartile range (IQR) are reported for continuous variables, whereas frequency and percentage (%) and 95% confidence intervals (CI) are reported for categorical variables.

For the comparison of continuous data, the nonparametric Mann-Whitney test was used. For the comparison of paired data, the Wilcoxon matched pairs test was performed. For analysis of categorical data, the Fisher’s exact test was applied.

Two-sided p-values less than 0.05 were considered significant. Statistical analyses were performed using SAS version 9.3 (SAS Institute Inc., Cary, NC, USA.) and GraphPad Prism version 5.04 (GraphPad Software, San Diego, CA, USA).

## Results

**Fig 1:**
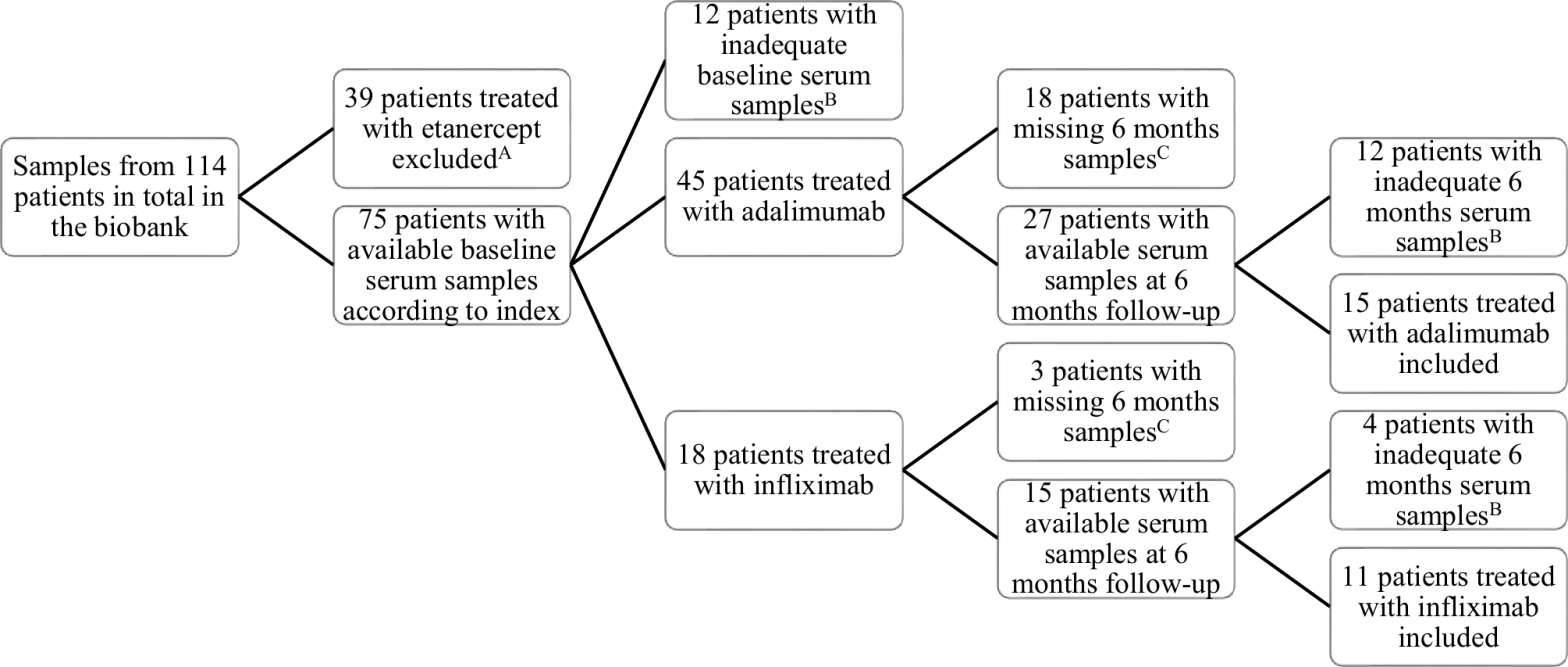
Flow diagram of the selection of patients in this study. Patients were recruited among all patients diagnosed with rheumatoid arthritis and treated with a TNFi, with serum samples available at the research biobank at the Parker Institute, Copenhagen University Hospital at Frederiksberg and Bispebjerg, Denmark. ^A^ Only patients treated with adalimumab or infliximab were included, as we did not have access to a reliable detection method regarding antibodies towards etanercept. ^B^ Vials were adequate only if they had not been previously thawed. At baseline, one vial of ≥ 3μl was required, and at 6 months follow-up 2 vials of ≥ 3 μl were required. ^C^ Missing samples due to inclusion in project not requiring 6 months sampling, termination of current treatment, or lost to follow-up.

**Fig 2:**
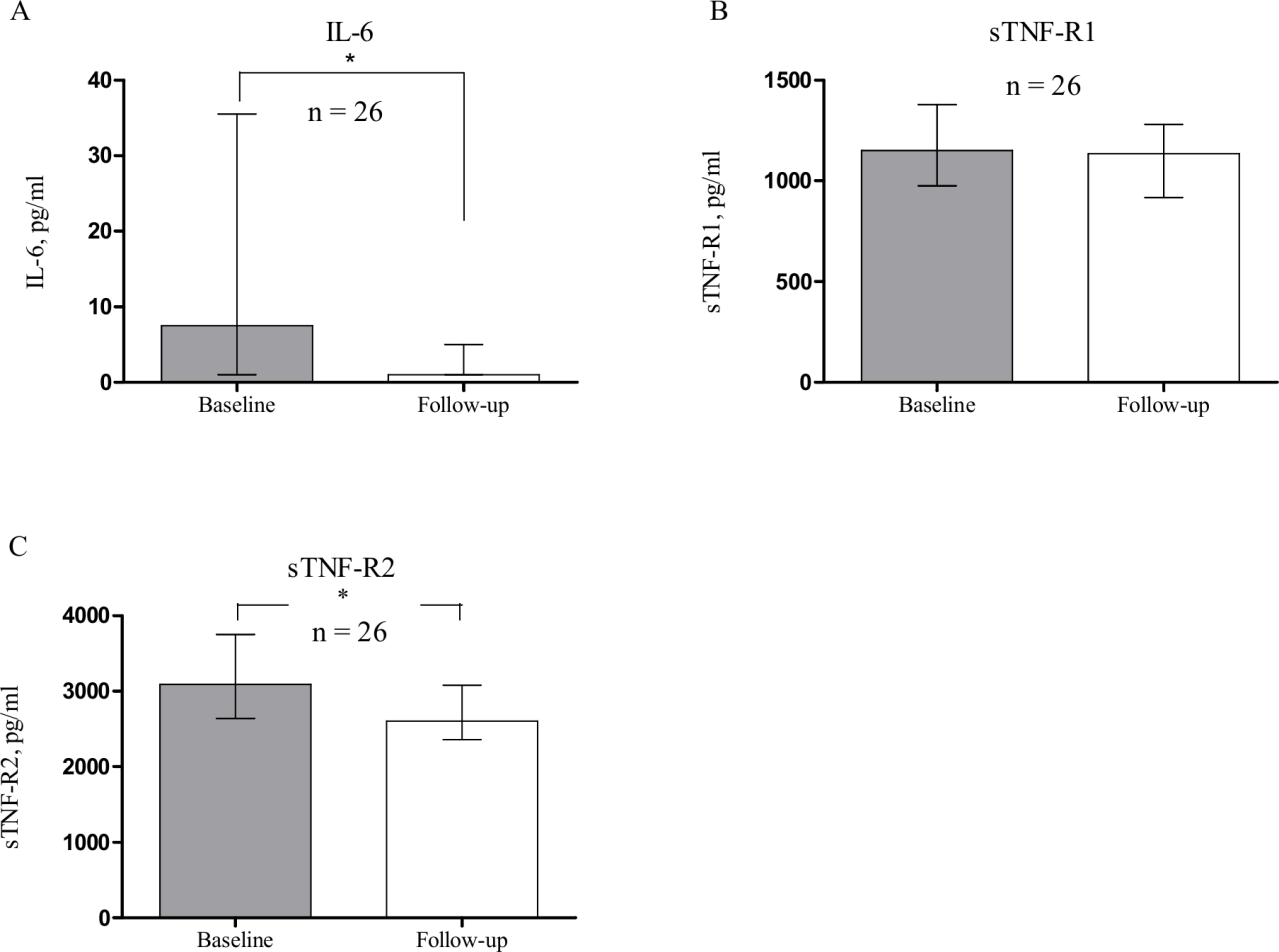
Changes in levels of IL-6, sTNF-R1 and sTNF-R2 following 6 months of treatment with adalimumab or infliximab. Level of IL-6 and sTNF-R1 and sTNF-R2 were determined in 26 patients with rheumatoid arthritis, 15 patients treated with adalimumab, and 11 patients treated with infliximab. Levels were determined prior to treatment (Baseline) and following 6 months of treatment (Follow-up). Levels of IL-6 (A) and sTNF-R2 (C) decreased significantly during treatment. *p < 0.05. Medians and interquartile ranges are shown.

A total of 26 patients with RA were included from the cohort (for the selection process, see **Fig 1)**. Fifteen patients were treated with adalimumab, and 11 patients were treated with infliximab (for baseline characteristics, see Table 1**)**. None of the participants were exposed to steroid treatment during the study, and only a few patients received sulfasalazine. The patients treated with adalimumab or infliximab did not differ in any of the baseline characteristics, neither did they differ in baseline levels of measured biomarkers. During the first 6 months of treatment with adalimumab or infliximab, DAS28(CRP) declined from 4.9 (4.2–5.7) to 3.5 (2.6–4.4) (*p* < 0.001) in the entire group, whereas the median level of CRP did not change significantly, from 5.0 (1.8–36)) mg/ml to 3.3 (1.4–9) mg/ml, respectively (*p* = 0.0535)). There was no difference in effect on DAS28(CRP),CRP, IL-6 or sTNF-R1 and -R2 between patients treated with adalimumab or infliximab. Both levels of IL-6 and sTNF-R2 decreased significantly during treatment (**Fig 2**). IL-6 decreased from 8 (1–36) pg/ml at baseline to 1 (1–5) pg/ml after 6 months treatment (*p* = 0.009). sTNF-R2 decreased from 3092 (2642–3750) pg/ml at baseline to 2604 (2361–3079) pg/ml at follow-up (*p* = 0.0071). Levels of sTNF-R1 did not change significantly during treatment from 1152 (976–1380) pg/ml to 1135 (919–1281) pg/ml (*p* = 0.2818). The levels of the other tested cytokines (TNF-α, IL-1b, IL-10, IL-17A, IL-17F, IL-33, TNF-β, IL-1ra, IL-8, IL-12(p70) and IL-13) were generally below detection limits of the kits (data not shown).

**Table 1:** Baseline characteristics of the 26 rheumatoid arthritis patients included in the study.

TNFi/ Characteristic	Adalimumab (n = 15)	Infliximab (n = 11)	Total (n = 26)
**Male gender, n (%)**	4 (27)	4 (36)	8 (31)
**Age, years**	53 (39–64)	54 (49–61)	54 (41–61)
**IgM-RF positive, n (%)**	11 (73)	8 (73)	19 (73)
**Previous biological DMARD, n (%)**	5 (33)	2 (18)	7 (27)
**Concomitant MTX, n (%)**	7 (47)	8 (72)	15 (58)
**MTX, mg/week**	17.5 (10–20)	17.5 (11.25–23.75)	17.5 (10–20)
**Disease duration, years**	5 (2–16)	6 (2–10)	6 (2–12)
**DAS28(CRP)**	5.0 (4.2–5.4)	5.3 (4.4–6.0)	5.0 (4.3–5.8)
**No. of tender joints**	8 (5–16)	8 (4–17)	8 (5–16)
**No. of swollen joints**	6 (2–8)	4 (2–7)	5 (2–8)
**CRP, mg/ml**	6.0 (2.0–34.0)	4.0 (1.0–36.0)	5.0 (1.8–36.0)
**IL-6 pg/ml**	8 (1–26)	7 (1–40)	8 (1–36)
**sTNF-R1 pg/ml**	1105 (946–1340)	1201 (1006–1481)	1152 (976–1380)
**sTNF-R2 pg/ml**	3100 (2777–3750)	2981 (2143–3750)	3092 (2642–3750)

* except when otherwise indicated, values are median (IQR); IgM-RF = IgM rheumatoid factor; DMARD = Disease-modifying antirheumatic drug; DAS28(CRP) = Disease activity score in 28 joints based on C-reactive protein (CRP); MTX = methotrexate.

### Levels of TNFi and development of anti-TNFi antibodies

Undetectable levels of TNFi were present in two patients treated with adalimumab (13% (2–39%)) and in four patients treated with infliximab (36% (14–64%). In patients with detectable levels of TNFi at follow-up, DAS28(CRP) and the level of IL-6 and sTNF-R2 decreased significantly during treatment, while this was not the case in the patients with undetectable TNFi (**Table 2**). Regarding the predictive value of baseline biomarkers, patients with undetectable levels of TNFi at follow-up had higher baseline levels of IL-6 (**Table 2**). The groups of patients with detectable and undetectable levels of TNFi did not differ in terms of age, gender, use of methotrexate, or the number of tender or swollen joints at baseline (data not shown), baseline DAS28(CRP), or baseline CRP (**Table 2**).

**Table 2:** Clinical variables and biomarkers according to whether TNFi was detectable in the patients’ blood following 6 months of treatment with adalimumab or infliximab.

•	• TNFi detectable (n = 20)	• TNFi undetectable (n = 6)	*• p*
• Concomitant MTX, n (%)	• 10 (50)	• 5 (83)	*• 0.197*
• MTX, mg/week	• 18.75 (15–21.25)	• 10 (7.5–21.25)	*• 0.214*
• DAS28(CRP)Baseline • Follow-up • *p*	• n = 18 • 4.8 (3.9–5.7) • 3.4 (2.6–4.4) • *<0.001*	• n = 6 • 5.1 (4.7–6.1) • 4.2 (3.0–5.2) • *0.156*	*•* *• 0.408* *• 0.183*
• CRP, mg/mlBaseline • Follow-up • *p* •	• n = 19 • 2 (1–13) • 2 (1–7) • *0.1243* •	• n = 6 • 36 (10–43) • 17 (5–31) • *0.5625* •	*•* *• 0.089* *• 0.014*
• IL-6, pg/ml • Baseline • Follow-up • *p*	• • 6 (1–22) • 1 (1–3) • *0.0143*	• • 44 (26–111) • 11 (1–38) • *0.3125*	*•* *• 0.031* *• 0.180*
• sTNF-R1, pg/ml • Baseline • Follow-up • *p*	• • 1152 (956–1369) • 1103 (861–1268) • *0.061*	• • 1197 (959–1998) • 1248 (1133–1650) • *0.844*	*•* *• 0.738* *• 0.073*
• sTNF-R2, pg/ml • Baseline • Follow-up • *p*	• • 3033 (2525–3663) • 2563 (2210–2892) • *0.011*	• • 3750 (2675–3750) • 2907 (2589–3265) • *0.3125*	*•* *• 0.165* *• 0.152*

*except when otherwise indicated, values are median (IQR); IgM- RF = IgM rheumatoid factor; MTX = methotrexate; DAS28(CRP) = Disease activity score in 28 joints based on C-reactive protein (CRP).

During the first 6 months of treatment with either of the TNFi, 6 out of 26 patients (23% (11–42%)) developed anti-TNFi Abs. Among these 6 patients, 4 had levels of TNFi below the detection limit. In the group of 20 patients without anti-TNFi Abs, 2 had undetectable levels of TNFi at follow-up.

**Fig 3:**
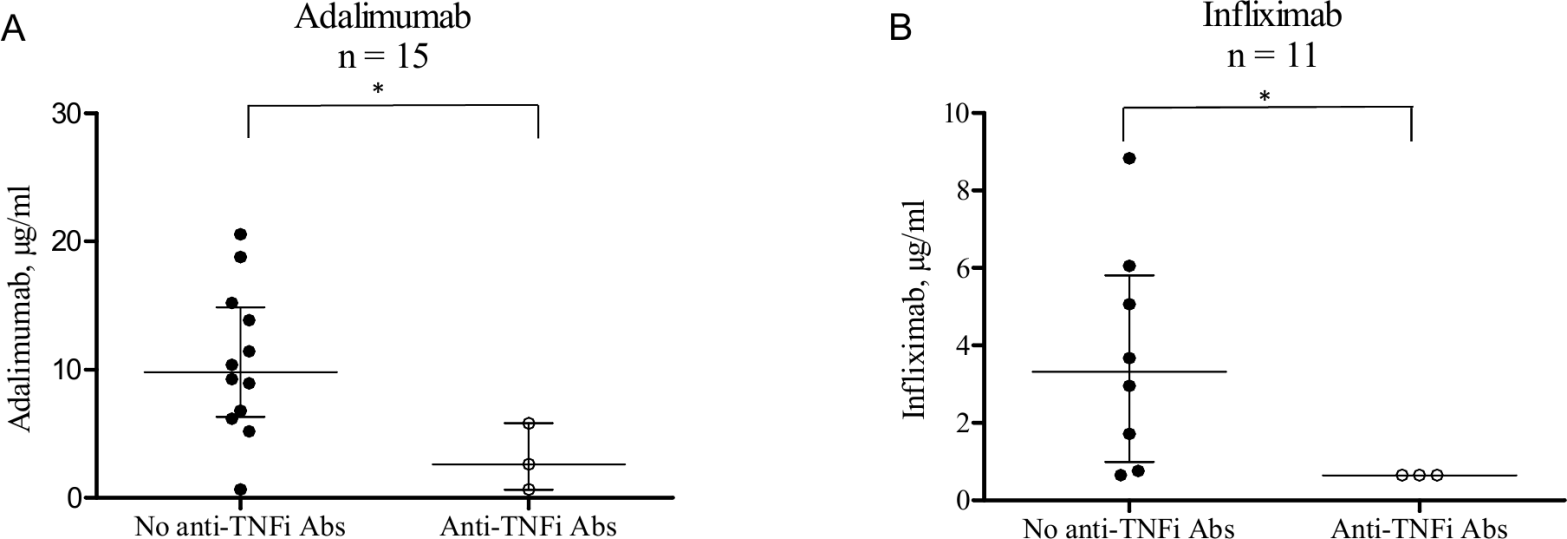
Serum TNFi levels in relation to antibodies against adalimumab and infliximab following six months of treatment. Antibodies towards the prescribed TNFi (anti-TNFi Abs) were measured in 26 patients with rheumatoid arthritis, using radioimmunoassay. Patients were treated with either adalimumab (A) or infliximab (B). Functional levels of TNFi were determined using reporter gene assays. Presence of anti-TNFi Abs correlated with lower levels of TNFi for both drugs *(*p* < 0.05). Medians and interquartile ranges are shown.

The development of anti-TNFi Abs was evenly distributed, with 3 out of 15 patients in the group treated with adalimumab (20% (6–46%) and 3 out of 11 patients in the group treated with infliximab (27% (9–57%). The concentration of TNFi for each of the drugs corresponded with anti-TNFi Ab status, as presence of anti-TNFi Abs was associated with decreased levels of adalimumab or infliximab (**Fig 3**).

In patients treated with adalimumab, median drug level was 2.6 (0.7–5.8) μg/ml in the patients with anti-TNFi Abs and 9.8 (6.3–14.9) μg/ml in patients without anti-TNFi Abs (*p* = 0.043). In patients treated with infliximab, all patients with anti-TNFi Abs had a drug level below detection limit of 0.7 μg/ml, while median level of drug was 3.3 (1.0–5.8) μg/ml in patients without anti-TNFi Abs (*p* = 0.037).

Absence of anti-TNFi Abs after 6 months of therapy was associated with a decline in DAS28(CRP) and a decline in levels of IL-6 and sTNF-R2, while levels of sTNF-R1 remained unchanged (**Table 3**). There was no significant drop in clinical disease activity score or in levels of IL-6 and soluble

**Table 3:** Variables according to development of anti-TNFi Ab production following 6 months of treatment with adalimumab or infliximab.

•	• Anti-TNFi Ab neg. (n = 20)	• Anti-TNFi Ab pos. (n = 6)	*• p*
• Concomitant MTX, n (%)	• 12 (60)	• 3 (50)	*• 1.00*
• MTX, mg/week	• 16.25 (11.25–20)	• 17.5 10–25.9)	*• 0.884*
• DAS28(CRP)Baseline • Follow-up • *p*	• n = 18 • 4.9 (4.0–5.7) • 3.4 (2.6–4.4) • *<0.001*	• n = 5 • 5.7 (4.4–6.6) • 4.0 (2.4–4.7) • *0.125*	*•* *• 0.152* *• 0.823*
• CRP, mg/ml • Baseline • Follow-up • *p*	• n = 18 • 4 (1–25) • 3 (1–9) *• 0.0729*	• n = 6 • 20 (3–43) • 9 (3–31) • *0.6741*	*•* *• 0.404* *• 0.130*
• IL-6, pg/ml • Baseline • Follow-up • *p*	• • 7 (1–25) • 1 (1–7) *• 0.020*	• • 33 (1–111) • 1 (1–22) • *0.316*	*•* • *0.323* • *0.602*
• sTNF-R1, pg/ml • Baseline • Follow-up • *p*	• • 1200 (956–1369) • 1135 (926–1277) • *0.220*	• • 1104 (978–1998) • 1160 (842–1650) *• 0.688*	*•* *• 0.879* *• 0.738*
• sTNF-R2, pg/ml • Baseline • Follow-up • *p*	• • 3092 (2525–3750) • 2583 (2210–2850) • *0*.*005*	• • 3213 (2639–3750) • 3087 (2671–3581) • *0.813*	*•* *• 0.829* *• 0.067*

*except when otherwise indicated, values are median (IQR); IgM- RF = IgM rheumatoid factor; MTX = methotrexate; DAS28(CRP) = Disease activity score in 28 joints based on C-reactive protein (CRP).

TNF receptors in patients with anti-TNFi Abs, and this group showed higher levels of sTNF-R2 at follow-up compared with patients without anti-TNFi Abs. Production of anti-TNFi Abs was not associated with age, gender, level of IgM-RF, concomitant methotrexate, disease duration, or the number of tender or swollen joints (data not shown). Nor was it associated with the baseline level of DAS28(CRP), CRP, IL-6, sTNF-R1 or sTNF-R2 (**Table 3)**.

## Discussion

Early development of anti-TNFi Abs in a cohort of RA patients and the impact of treatment on readily available soluble biomarkers were investigated in this study. As expected, treatment with TNFi had an overall impact on disease activity by lowering DAS28(CRP). To our surprise, however, we did not find a general decrease in the levels of CRP during treatment, and we ascribe this to our low baseline CRP. In contrast, TNFi therapy resulted in reduced circulating levels of IL-6, which is likely to reflect the drug-induced decrease in inflammatory activity in accordance with previous findings [24;25].

To our knowledge, the association of TNFi therapy with lowered circulating levels of sTNF-R2 has not previously been demonstrated, although others have tried [15;26]. Genome sequencing have found that mutations in TNF-R2 are associated with decreased response to TNFi treatment [27], which suggests that clinical effect of TNFi could be mediated, at least in part, by TNF-R2. *In vitro*, infliximab increases the release of TNF-R2 from human monocytes and reduces the expression of the receptor [28]. These actions increase the TNF- α neutralizing activity and lower the cellular response to TNF-α. The receptor shedding and the decreased expression may explain the decrease in circulating sTNF-R2 seen following infliximab treatment, but also the observed cytotoxic effect of infliximab and adalimumab on TNF-α expressing cells may contribute to the lowered levels of sTNF-R2 [29;30].

The frequency of anti-TNFi Ab-positivity was 23%, corresponding to previous findings ranging from 19–44% [4;11;12]. Although not statistically significant, the observed difference in immunogenicity between adalimumab (20% anti-TNFi Ab-positive) and infliximab (27% anti-TNFi Ab-positive) are in accordance with previous observations, in which infliximab appears slightly more immunogenic than adalimumab [4;6;11;12;31–35]. Previous studies have shown that patients with high baseline disease activity [11] or lower levels of TNFi early in the treatment course [4] have increased risk of developing anti-TNFi Abs. We were not able to corroborate such an association and found no associations between baseline characteristics or biomarkers and presence of anti-TNFi Abs. However, as previously shown, the presence of anti-TNFi Abs correlated with simultaneous low or undetectable drug levels and with an inability to decrease clinical disease activity [4;6;11;12;34]. These impaired drug levels were preceded by higher baseline levels of IL-6 and a trend towards higher baseline CRP.

A few remarks on the strengths and limitations of this study must be made. Sera were always sampled when drug levels were at their lowest (trough) in a treatment cycle, and sera used for biomarker analysis were meticulously prepared in order to preserve the biomarkers in the best way possible. Centrifugation was initiated instantly following sedimentation, sera were frozen immediately after preparation, and samples used for the analyses were not previously thawed. As most of the out-of-range values for sTNF-R2 were from baseline samples, the inability to determine precise concentrations of high levels of the receptor may have underestimated the impact of TNFi on this biomarker. Although the higher baseline levels of CRP in patients who later had impaired drug levels did not significantly differ from the rest, a *p*-value of 0.089 in this exploratory study calls for attention as the finding may reflect a type 2 error due to a limited study population. As previously explained, development of anti-TNFi Abs occur most rapidly during the first 6–12 months of therapy. We may have underestimated the immunogenicity of the two TNFi in this study by choosing the 6-month follow-up. Pragmatically, this was our best option, as widening the time frame to 12 months would have limited our sample size considerably.

## Conclusion

Our findings underline that anti-TNFi Abs have an impact on levels of TNFi and we further emphasize the impact TNFi levels have on modulating inflammatory activity and ensuring clinical efficacy. Finding that increased baseline levels of IL-6 are associated with undetectable levels of TNFi after 6 months of therapy suggest that in patients with a higher inflammatory load at baseline, treatment should be intensified early on, to ensure adequate disease control. Attention to baseline inflammatory activity may foresee later treatment failure by inadequate drug levels and may promote individualized treatment regimens, improving the clinical outcome for the patient. The results from this small-scale study should be confirmed in larger cohorts.

## Supporting Information

S1 Table (XLSX)
